# Simultaneous Laser Reduction of Sn/Sb Salts and Graphene Formation as Innovative Anode Material for Li‐ and Na‐Ion Batteries

**DOI:** 10.1002/gch2.202500356

**Published:** 2026-04-29

**Authors:** Vincenzo Vezzoni, Michele Setti, Giacomo Magnani, Laura Fornasini, Silvio Scaravonati, Alessia Rinaldi, Niyaz Ahmad, Lorenzo Pasetti, Danilo Bersani, Giovanni Bertoni, Michele Sidoli, Gioele Genovese, Mauro Riccò, Daniele Pontiroli

**Affiliations:** ^1^ Nanocarbon Laboratory cIDEA & Department of Mathematical Physical and Computer Sciences University of Parma Parma Italy; ^2^ GISEL National Centre of Reference for Electrochemical Energy Storage Systems INSTM National Interuniversity Consortium of Materials Science and Technology Firenze Italy; ^3^ Department of Mathematical Physical and Computer Sciences University of Parma Parma Italy; ^4^ CNR – Istituto Nanoscienze Modena Italy

**Keywords:** antimony, laser‐induced graphene, lithium‐ion batteries, sodium‐ion batteries, tin

## Abstract

The increasing demand for portable electronics and electric vehicles has made the development of advanced electrochemical energy storage systems essential. Lithium‐ion batteries (LIBs), which predominantly use graphite anodes, face limitations in capacity and performance at high current rates. As a result, alternative anode materials such as tin (Sn) and antimony (Sb) have gained attention for both LIBs and sodium‐ion batteries (SIBs) as well, due to their high theoretical capacity. However, their practical application is hindered by significant volume expansion during cycling, leading to electrode degradation. This study presents a novel approach to improve the stability and performance of Sn and Sb anodes by incorporating them into a laser‐induced graphene (LIG) matrix. LIG was synthesized via laser ablation of a polyimide precursor mixed with metal‐salt precursors, directly onto a copper current collector, enabling the in situ formation of Sn and Sb metallic nanoparticles (NPs) and SnSb alloy NPs, embedded in a few graphene layers. The localized high‐temperature generated by the laser facilitated nanoparticle formation while simultaneously creating a protective carbon shell around the NPs, mitigating volume expansion and enhancing electrochemical stability. Electrochemical testing demonstrated that the LIG‐metal composites exhibited superior performance compared to bare LIG in both LIB and SIB. LIG‐Sn composite achieved the specific capacity of 380 mAh g^−1^ in LIBs and 155 mAh g^−1^ in SIBs after 80 and 50 cycles, respectively. These results highlight the potential of LIG‐based Sn and Sb composites as scalable, binder‐free anode materials for next‐generation rechargeable batteries.

## Graphical abstract

A novel, scalable method for laser‐induced graphene–metal composite anodes for Li‐ion and Na‐ion batteries fabrication is reported. Embedded Sn, Sb, and Sn‐Sb alloy nanoparticles in graphene are created, which can accommodate volume changes during cycling, enhancing stability and rate performance. The optimized Sn composite features 5 nm particles and delivers a reversible capacity of 624 mAh g^−^^1^ in Li‐ion half‐cells.

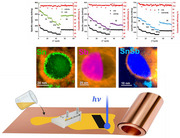

## 1 Introduction

Inspired by the increasing demand for cheaper, safer, and scalable energy storage devices, research has explored novel materials for the next‐generation of materials for battery applications [1]. Indeed, rechargeable batteries stand out as the primary technology for storing electrical energy through reversible chemical reactions. Secondary batteries can accumulate energy from external sources and release it cyclically with efficiencies approaching 100% [2].

Lithium‐ion batteries (LIBs) dominate the energy storage landscape, valued for their high energy density and power. Initially developed for portable electronics, LIBs are now pivotal in the automotive sector, driving the transition to electric vehicles. Meanwhile, alternative battery chemistries based on more abundant alkali metals such as sodium [3], potassium [4], and magnesium [5] are emerging. Sodium‐ion batteries (SIBs), for instance, offer a promising low‐cost and environmentally friendly alternative to LIBs. However, the larger Na ionic radius (1.02 Å compared to that of lithium at 0.76 Å) prevents the applicability of traditional LIB materials and results in comparatively lower capacity and power density.

Currently, LIBs rely on well‐established materials, consisting in graphitic carbon as the anode and metal oxides or phosphates, like LiCoO_2_, Li(Ni_x_Mn_y_Co_z_)O_2_ with *x+y+z = 1*, and LiFePO_4_ as the cathodes [6, 7, 8]. Focusing on anodic materials, graphite has a theoretical capacity of 372 mAh g^−1^, with practical values dropping to ∼300 mAh g^−1^ [1, 9], particularly under high charge/discharge rates. Furthermore, graphite is unsuitable for SIBs, offering a capacity of merely 30 mAh g^−1^ due to the inability of Na‐ions to intercalate effectively between graphite layers [10].

To overcome these limitations, considerable interest has shifted to group 13–15 metals, which can form intermetallic alloys with lithium and sodium, offering significantly higher theoretical capacities. For example, tin (Sn) displays 994 mAh g^−1^ in LIBs [11, 12, 13] and 894 mAh g^−1^ in SIBs [14, 15, 16], while antimony (Sb) provides 660 mAh g^−1^ for both systems [17, 18, 19, 20], far surpassing the capacity of graphite. Additionally, Sn, Sb, and SnSb alloys exhibit high electrical conductivity, facilitating fast charge/discharge cycles without requiring conductive additives, and are relatively abundant in the Earth's crust.

Despite their advantages, these materials face a major challenge, namely the significant volume expansion during charge and discharge processes. For example, the lithiation of Sn leads to the formation of intermetallic compounds, such as Li_22_Sn_5_ (the maximum lithiated form), resulting in a volumetric expansion of up to 260%. Similarly, sodiation forms compounds like Na_15_Sn_4_, with expansions reaching 420%. These dramatic volume changes cause particle pulverization and rapid capacity degradation over cycling.

To mitigate this issue, strategies such as nanosizing the active materials and employing structural templates have been explored [11, 19, 20, 21]. Haoze Song et al. [22]. demonstrated that the capacity retention of Sn‐based anode is strongly correlated with the particle size of Sn in the electrode, with smaller particles leading to improved performance. In addition, carbon‐based nanostructures such as graphene, carbon nanotubes, and amorphous carbon can serve as templates to buffer volume changes and prevent particle degradation as reported in the literature [23]. However, traditional synthesis methods often require multiple processing steps, hazardous reagents, and high costs, limiting their scalability.

Recently, a novel and scalable method for graphene synthesis, known as laser‐induced graphene (LIG), has gained significant attention [24, 25]. This technique involves the laser irradiation of polymer substrates, such as polyimide (PI), to produce graphene directly without requiring high‐temperature furnaces or toxic chemicals. LIG has demonstrated considerable potential as an anode material for rechargeable batteries [25, 26, 27], including LIBs and SIBs. Its performance, however, can be further improved by incorporating active materials from groups 13–15 [28].

Numerous studies have explored the decoration of LIG with heteroatoms or heterostructures [29, 30, 31, 32]. However, many of these approaches involve multiple processing steps, often requiring the separate preparation of nanosized crystals prior to LIG production. Additionally, the interaction of light with matter during LIG synthesis remains not well understood, particularly in the context of one‐step methods for heterostructure formation. For example, some studies report the formation of metal oxides during synthesis [30, 31], while others describe the production of metallic nanoparticles (NPs) [33, 34, 35], highlighting the need for further investigation to clarify these mechanisms.

In this work, we present a simple and cost‐effective one‐step method to produce composite anode materials combining LIG with metallic Sn, Sb, and SnSb alloys, which can potentially overcome the currently used graphitic material for LIB and open the possibility for their use in commercial SIB. This process involves direct carbonization of PAA and simultaneous in situ reduction of metal precursors, yielding metallic NPs embedded within few‐layer graphene. The resulting materials exhibit high specific capacities and excellent cycling stability, making them compelling candidates for next‐generation LIBs and SIBs.

## 2 Experimental

### 2.1 Synthesis of LIG‐Sn, LIG‐Sb, and LIG‐SnSb Composites

SnCl_2_ (>99.99%), Sb (CH_3_CO_2_)_3_ (>99.99%), *N*‐methyl‐2‐pyrrolidone (NMP, anhydrous, 99.5%), and polyamic acid solution (poly(pyromellitic dianhydride‐co‐4,4′‐oxydianiline), 12.8 wt.% in 80% NMP/20% aromatic hydrocarbon) (PAA) were purchased from Merck KGaA. Copper foil, 12 µm thick and ≥ 99.9% purity, was purchased from Tmax Battery Equipment.

For the lithium half‐cell assembly, CR2032 coin cell cases were used. Lithium metal disks were employed as a negative electrode. Polyvinylidene fluoride (PVDF, Solvay, Solef 5310) was used as the separator, and 1.0 m LiPF_6_ in EC/DMC (50/50 v/v, Sigma‐Aldrich, battery grade, < 15 ppm H_2_O, < 50 ppm HF) served as the electrolyte.

For the sodium half‐cell assembly, the same CR2032 coin cell cases were used. Metallic sodium disks were prepared by shaping sodium metal (Merck, 99.9%) on a stainless‐steel spacer. Glass fiber was used as the separator, and 1.0 m NaPF_6_ (Merck, 99.9%) in EC/DMC (50/50 v/v) was freshly prepared in the laboratory as the electrolyte.

**FIGURE 1:**
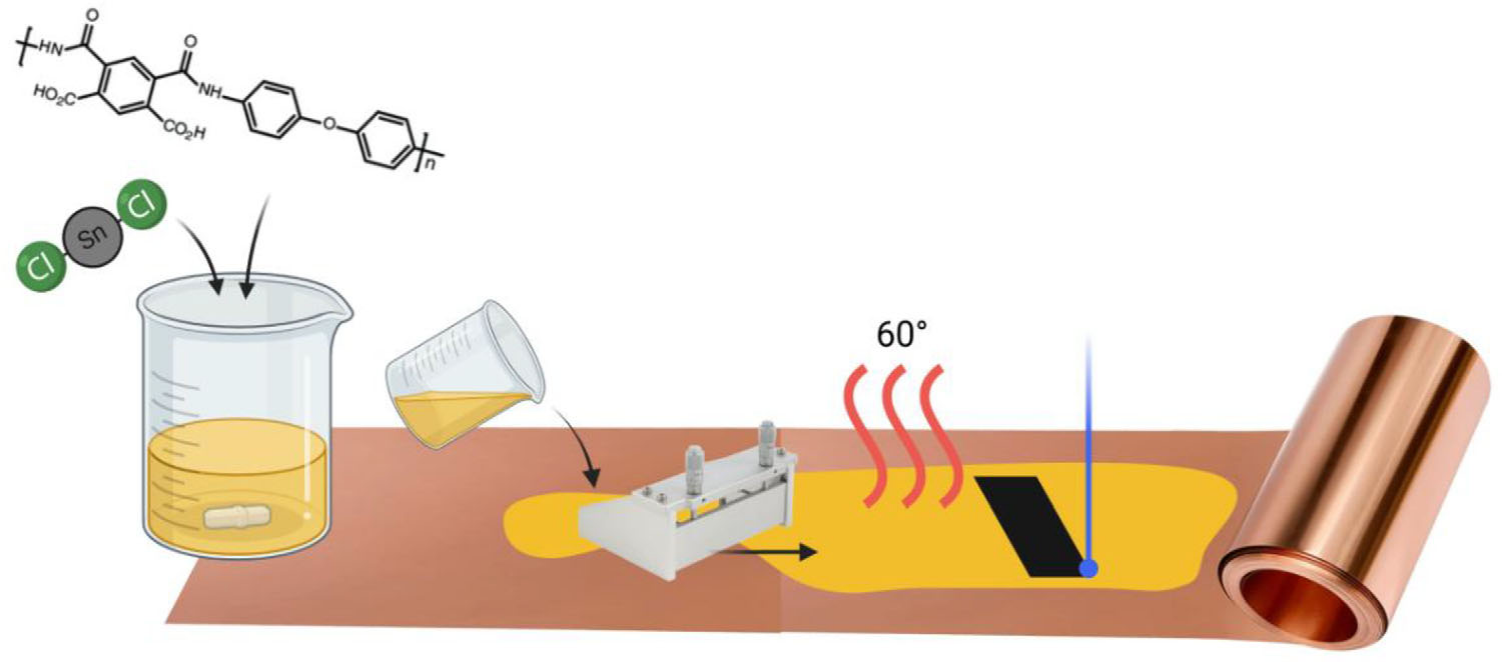
Schematic procedure of LIG‐metal composites realization. First, the solution containing PAA and metal salt precursor is prepared. Second, the solution is cast on copper foil and dried at 60°C, and finally treated with the blue laser.

The one‐step synthetic method, schematically reported in Figure 1, involved preparing metal salt solutions in 3 mL of PAA, resulting in the samples LIG‐Sn, LIG‐Sb, and LIG‐SnSb, respectively. For the LIG‐Sn sample, 417.7 mg of SnCl_2_ powder was dissolved in the PAA solution until the solution became transparent. For the LIG‐Sb sample, 647.6 mg of Sb(CH_3_CO_2_)_3_ was first ground in a mortar, dissolved in 1 mL of NMP, and then added to the PAA solution. Since Sb(CH_3_CO_2_)_3_ is poorly soluble in most organic solvents, the resulting solution was not fully transparent but appeared light yellow. For the LIG‐SnSb sample, 196.4 mg of SnCl_2_ and 311.8 mg of Sb(CH_3_CO_2_)_3_ were dissolved in 1 mL of NMP and then added to 3 mL of PAA.

All solutions were stirred overnight using a magnetic stirrer and then treated in an ultrasonic bath for 30 min. If the final solution was too viscous to cast, NMP was added drop by drop to adjust its rheology. The prepared solutions were cast onto copper foil current collectors using a doctor blade set to a thickness of 150 µm. The slurry‐coated copper foil was cured in a vacuum oven at 60°C for 4 h to evaporate the solvent completely.

The coated films, comprising PAA mixed with metallic salts, were then treated using a 3D printer equipped with a laser module (SnapMaker Original modular 3D printer) to simultaneously carbonize the polymer into graphene and decompose the metal salts, forming nanoparticles embedded in few‐layer graphene. The laser cutting machine was equipped with a diode laser source (λ = 450 nm) with a maximum power of 1600 mW.

Laser conversion parameters were optimized to minimize the resistance between the active material and the copper current collector, ensuring complete conversion of the precursor material into LIG and achieving good contact between the active material and the metallic substrate (Figure S1). The laser head was set at the focal distance of Z = 42 mm over the sample, and the speed and power were varied to minimize the resistance. The optimal parameters were determined to be a laser power of 90% (1440 mW) and a scanning speed of 500 mm/min with a double passage of the laser. The laser operated in raster mode with a spacing of 0.05 mm between filling lines, enabling the creation of customized electrode shapes. For this study, large rectangular electrodes (2 × 5 cm^2^) were printed with the laser to facilitate cutting and assembly. The as‐obtained LIG electrodes were cut into disks of 16 mm or diameter, pressed under the hydraulic press at 2 MPa, and dried in the oven at 60°C for the overnight. At the end, they were assembled and cycled in a half‐cell configuration, with LIG‐metal composites at the positive electrode and metallic lithium or sodium at the counter electrode.

## 3 Methods

The surface morphology of LIG‐metal composites was analyzed using a Hitachi TM4000Plus II tabletop scanning electron microscope (SEM) from Hitachi Co., Japan. The SEM was operated at an accelerating voltage of 15 kV, with backscattered electron imaging conducted at magnifications ranging from 2000× to 10000×. This method enabled a thorough examination of the surface features of the metal‐decorated LIG samples and the determination of their elementary composition.

Room‐temperature PXRD analysis of the LIG‐metal composites samples was performed using a Bruker D8 Discover diffractometer in Debye–Scherrer geometry. The instrument was equipped with a Cu anode (CuKα radiation) as the X‐ray source, a Göbel mirror, and a 0.5 mm collimator. Diffraction patterns were recorded with a Rayonix MX225 2D area detector, featuring 73.2 µm pixels for high‐resolution data, positioned 110 mm from the sample. To reduce preferential orientation effects, the samples were sealed in 0.5 mm glass capillaries and spun during data collection. Silicon was used as the calibration standard for the detector. The collected diffraction images were processed using FIT2D software to extract powder patterns, which were further refined using the Rietveld method with the GSAS‐II suite. The Quantitative Phase Analysis (QPA) has been performed without internal reference, and the relative weight fraction has been calculated according to Equation (1):

(1) $$W_{A}=\frac{k_{A}A_{A}}{k_{A}A_{A}+k_{B}A_{B}}\;\mathrm{and}\;W_{B}=\frac{k_{B}A_{B}}{k_{A}A_{A}+k_{B}A_{B}}\;$$

where *A_A_* and *A*_B_ correspond to the integrated intensities of the two phases, while *k*_A_ and *k*_B_ correspond to the optimized parameters during the Rietveld refinement.

The morphology of the samples was examined using a Thermo Fisher Scientific Talos F200S transmission electron microscope (TEM), equipped with a Schottky gun electron source operating at 200 kV, capable of achieving an information limit of 0.12 nm in high‐resolution images (HRTEM). The materials were detached from the substrate to obtain a powder form, which was then dispersed in isopropanol to create suspensions. Small volumes from the suspensions were carefully deposited onto the holey carbon films of the copper TEM grids and dried thoroughly. Once prepared, the samples were loaded into the microscope for examination. Spatial chemical compositions were obtained through electron energy‐loss spectroscopy (EELS) in scanning mode (STEM) at a camera length of 60 mm (High‐Angle‐Annular‐Dark‐Field or HAADF) with a post‐column spectrometer (Gatan Inc.). The average compositions of the samples were verified with energy‐dispersive X‐ray spectroscopy (EDXS). The combination of TEM with EELS and HAADF provides a comprehensive analysis of LIG‐metal composites.

Raman measurements were obtained using a confocal micro‐spectrometer, the Horiba Jobin Yvon LabRam (HORIBA Scientific, Kyoto, Japan), combined with an Olympus BX40 microscope with a 50× ULWD objective yielding a spatial resolution of approximately 2 µm. The instrument featured an 1800 grooves/mm grating allowing a spectral resolution of 4 cm^−^^1^, an XY motorised stage, and a Peltier‐cooled silicon CCD. The excitation source employed was a 473.1 nm frequency‐doubled Nd:YAG laser. Spectra were measured throughout the range of 100–2000 cm^−^^1^, to investigate the defectivity of the carbon by comparing the intensities of the D and G bands and to evaluate the presence of nanoparticles, and up to 3000 cm^−1^ to analyze the 2D band of graphene. Calibration of the system was achieved by reference the 520.7 cm^−^^1^ Raman peak of silicon. Typically, data acquisition was performed for 30 s and 3 repetitions. The LabSpec 5 software was employed for data analysis.

The galvanostatic charge–discharge (GCD) analysis was performed to evaluate the capacity, efficiency, and capacity retention of the half cells. The measurements were carried out using a Landt CT2001A and a Neware BTS‐4008 (5 V, 50 mA) battery testing system. GCD measurements were conducted under both constant current (chronopotentiometry).

## 4 Results and Discussion

The synthesis method reported in this work enables the direct preparation of graphene‐based anodic materials on a copper collector under ambient conditions, without the need for a binder. The high energy delivered by the laser source simultaneously converts the PAA substrate into graphene [24, 25] and decomposes the metal salt precursors into metallic nanoparticles (NPs). The active material was scraped from the copper collector and analyzed via PXRD measurements, sealed in a glass capillary to confirm the conversion of the precursors.

**FIGURE 2:**
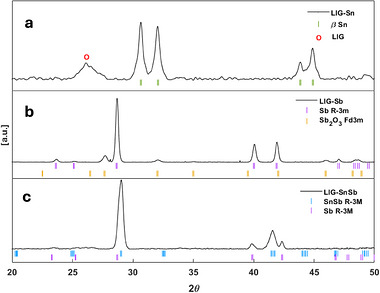
PXRD pattern of (a) LIG‐Sn, (b) LIG‐Sb, and (c) LIG‐SnSb. In figure (a), the peaks are associated to the β‐Sn structure of metallic Sn, while the large band at 2θ = 26° is ascribed to the LIG signal. In (b), the main phase is indexed by the Sb(0) phase, however traces of Sb_2_O_3_ are detected. In (c) the crystallisation of the SnSb phase is confirmed. The main peaks come from this phase; however, spurious peaks are ascribed to metallic Sb phase formation.

For the LIG‐Sn sample, the PXRD (Figure 2a) pattern revealed primarily the formation of metallic Sn‐NPs. Peaks at 2θ = 30.64°, 32°, 43.86°, and 44.9° correspond to the tetragonal β‐Sn phase of metallic tin (PDF 00‐004‐0673) [11, 14], while a broad band at 2θ = 26° was attributed to the (002) stacking direction of graphite [36, 37], indicating the presence of few stacked graphene layers characteristic of LIG. The broad diffraction features associated with β‐Sn suggest nanoscale crystallite dimensions. Using the Scherrer equation during Rietveld refinement, which incorporated anisotropic size broadening (Rwp = 9%), the mean particle size of β‐Sn NPs was estimated at 38 ± 3 nm.

The formation of pure metallic tin clearly demonstrates the reduction of Sn^2^^+^ precursors to the metallic phase, following the proposed reaction mechanism:

(2) $$Sn^{2+}+C+O_{2}\overset{h\nu }{\to }\mathrm{Sn}+CO_{x}$$

The high localized temperatures achieved by the laser, combined with the absence of oxygen during LIG formation (as CO_2_ is generated in situ), facilitate the carbothermal reduction of SnCl_2_, yielding Sn‐NPs as summarized in Equation (2).

For the LIG‐Sb sample, PXRD analysis (Figure 2b) confirmed the formation of metallic Sb nanoparticles, with characteristic peaks at 2θ = 23.7°, 25.1°, 28.7°, 40°, 41.9°, 47°, 48.7°, and 51.5°, corresponding to the rhombohedral Sb phase (PDF 00‐085‐1322) [17, 21]. However, additional broad features at 2θ = 27.7°, 32°, 35°, 45.9°, and 54.5° were identified as the cubic phase of Sb_2_O_3_ (PDF 00‐005‐0534) [38].

Quantitative phase analysis (QPA) using Rietveld refinement (Rwp = 5.5%) indicated that the sample consisted of 88% metallic Sb and 12% Sb_2_O_3_ by weight (Figure S3). The mean particle sizes, calculated via the Scherrer equation with anisotropic broadening, were 79 ± 4 nm in the *x*‐*y* plane and 33 ± 2 nm along the *z*‐axis for the Sb‐crystallites, suggesting a nanoplateled structure. Despite the partial oxidation, the majority of metallic Sb content supports a carbothermal reduction mechanism in Equation (3):

(3) $$Sb^{3+}+C+O_{2}\overset{h\nu }{\to }Sb^{0}+CO_{x}$$

For the LIG‐SnSb sample, PXRD analysis confirmed the formation of the SnSb alloy in a rhombohedral stistaite (Figure 2c) structure (PDF 00‐033‐0118), with peaks at 2θ = 29.1°, 41.6°, and 51.8° [39, 40]. Additional features, such as a shoulder at 2θ = 28.7° and peaks at 39.9° and 42.3°, indicated the presence of free metallic Sb within the matrix, consistent with the rhombohedral structure observed in the LIG‐Sb sample. The segregation of metallic Sb likely resulted from the non‐uniform distribution of Sb(CH_3_CO_2_)_3_ in the precursor solution, stemming from its limited solubility.

Rietveld refinement (Figure S4) performed in the 2θ range of 28°–53° (Rwp = 4.6%) quantified the sample composition as 91% SnSb and 9% Sb by weight. The average particle size of SnSb‐NPs, determined from peak broadening, was calculated to be 46.1 ± 2.2 nm. The reaction mechanism for SnSb formation during synthesis is proposed in Equation (4):

(4) $$Sn^{2+}+Sb^{3+}+C+O_{2}\overset{h\nu }{\to }\mathrm{SnSb}+CO_{x}$$

The PXRD results confirm the successful one‐step synthesis of LIG‐based anode materials, demonstrating the laser's ability to simultaneously carbonize the polymer and reduce metal salts into active nanoparticles or alloys in the nanoscale domain. From our knowledge, this is the first time that composites with Sb and SnSb NPs and LIG are obtained. Moreover, we demonstrate the possibility to obtain not only single metallic phases but also the formation of metal‐alloys, opening the possibility for future investigations of novel composite synthesis.

### 4.1 Morphology Characterization

**FIGURE 3:**
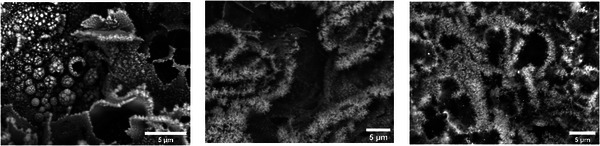
SEM images of LIG‐Sn (a), LIG‐Sb (b), and LIG‐SnSb (c) display the classical morphology of LIG, completely covered by metallic NPs. Larger particles tend to concentrate on the edges of the graphene structure.

SEM morphological analysis was performed on LIG‐metal composites using Hitachi TM4000Plus as described in previous session. The large quantity of metallic atoms in the matrix of the synthesized composites greatly influences the morphology of the sample. Metal NPs appear to completely cover the carbon matrix, giving the flakes a shiny appearance due to the high Z‐contrast from the Sn and Sb atoms. Many of the NPs tend to aggregate into larger clusters, as observed in Figure 3. These larger particles tend to form at the edges of the graphene, where probably the high number of defects catalyze their nucleation. In the case of LIG‐Sn, the nanoparticles appear more spherical in shape, completely covering the graphene flakes. On the other hand, LIG‐Sb mostly presents a high concentration of elongated structures along the edges of the graphene flakes and more spherical on top of the flakes. Differently, LIG‐SnSb appears less uniform than the previous samples, and also in this case, elongated structures are present at the edges.

EDX analysis of LIG‐Sn and LIG‐SnSb in Figure S5 confirms the presence of Sn and carbon, as well as a small amount of chlorine, suggesting that not all the SnCl_2_ precursor has been fully decomposed during the laser treatment. As a result, some SnCl_2_ remains electrochemically inactive in the electrode. Such findings suggest that also not all Sb(CH_3_CO_2_)_3_ may be completely decomposed during laser conversion. On the other hand, it is well known that not all the PAA polymeric fraction is converted, as the remaining polymer allows the mechanical stabilization of the electrode on the collector and prevents it from cracking. This seems to be confirmed by the relatively large amount of oxygen (15% atomic) in the EDX spectrum (Figure S5).

The formation of NPs in the LIG‐Sn, LIG‐Sb, and LIG‐SnSb samples was further confirmed by TEM analysis, providing complementary evidence to the PXRD results. High‐resolution imaging and Fourier Transform (FFT) analysis verified the presence of metallic nanoparticles in the LIG‐Sn and LIG‐Sb samples and confirmed the formation of the SnSb alloy in the LIG‐SnSb sample.

**FIGURE 4:**
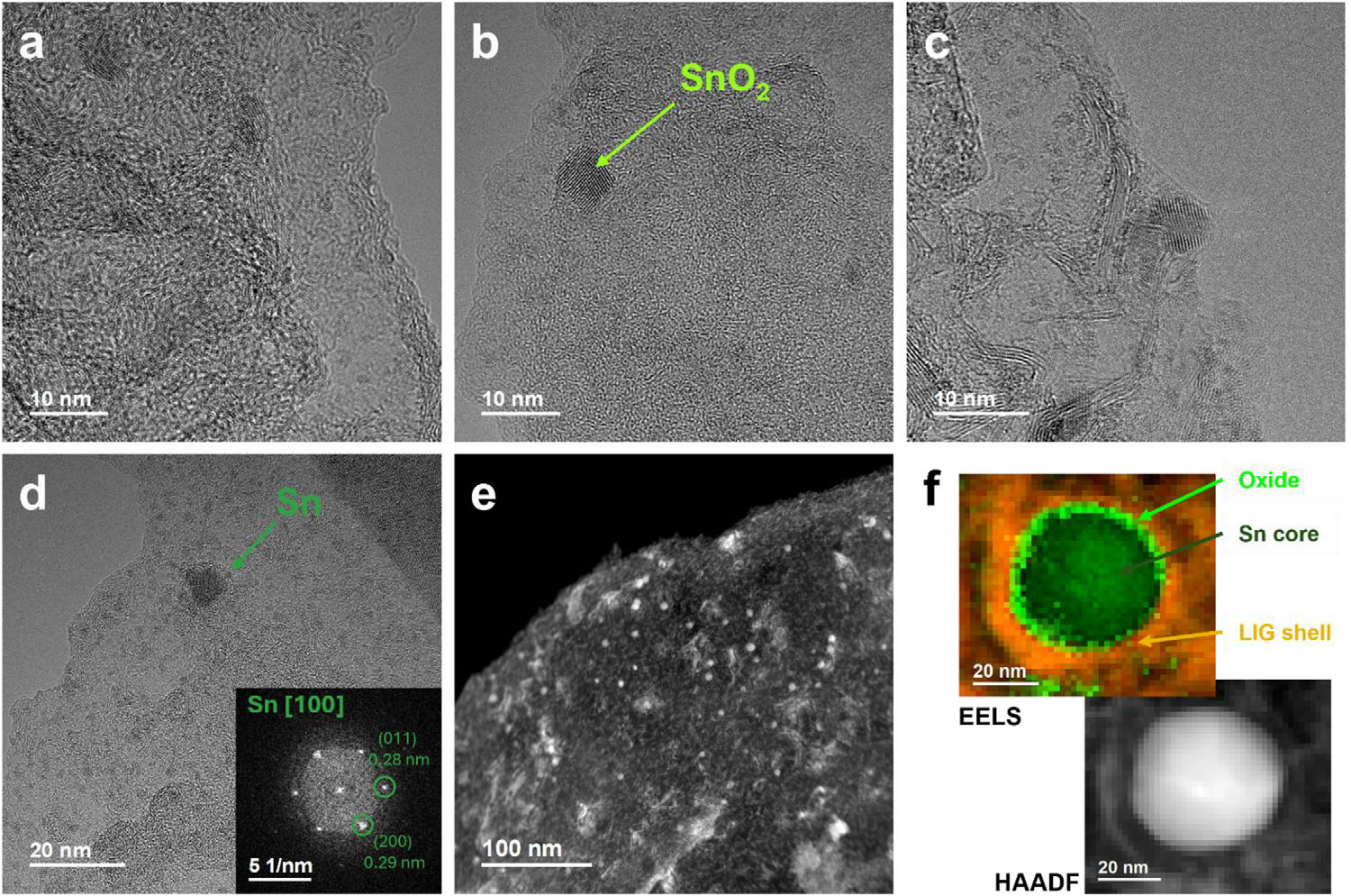
TEM analysis of LIG‐Sn sample. In HRTEM mode, the figures (a–d) show the very small dimensions of Sn‐NPs in the sample, in many cases lower than 2 nm. From figure (c), a particle of 5 nm is appreciable, surrounded by a thin layer of graphene. In figure (e) is reported the HAADF image of the sample, evidencing a distribution of heavy atoms all over the sample. (f) The EELS map of Sn particle is reported, evidencing a core–shell structure composed of a nucleus made of Sn(0) (dark‐green) and a thin shell of SnO_2_ (light‐green). In orange it is reported a thin layer of carbon from LIG, which protect the particle from complete oxidation and ensures a good anchor to the graphene backbone.

The atomic resolution achieved by High‐resolution Transmission Microscopy (HRTEM) revealed in LIG‐Sn a population of nanoparticles with diameters predominantly below 2–3 nm (Figure 4a–d), including clusters comprising only a few atoms, as shown in Figure 4b. While PXRD analysis estimated an average nanoparticle size of approximately 38 nm, the TEM observations highlight a wider size distribution, including numerous smaller particles below the detection limit of diffraction‐based techniques. These nanoparticles display a nearly spherical morphology and are coated with a carbon layer. Even HAADF in Figure 6e displays a wide distribution of Sn‐atoms on the carbon matrix, consisting in an almost uniform covering of the graphene sheet, underline the formation of small particles.

Spatially resolved EELS imaging further elucidated the chemical structure of the nanoparticles. The analysis confirmed that the Sn‐NPs comprise a metallic tin core encased within a thin shell of tin oxide (SnO_2_), itself surrounded by a few layers of graphene (Figure 4f). To enhance chemical mapping precision, the EELS dataset was subjected to principal component analysis (PCA) for noise reduction, followed by K‐means clustering to robustly classify the dominant chemical species, thereby confirming the core‐shell configuration.

The presence of SnO_2_ shell on the metallic core is likely attributable to incomplete graphene encapsulation, enabling surface oxidation during air exposure due to the ambient synthesis conditions. Nevertheless, the bulk of the nanoparticles retains its metallic character. Moreover, the high power of the laser employed during synthesis may have dual effects: while it effectively drives carbothermal reduction, it may also interfere with the formation of a uniform graphene shell or prevent its full development on the surface of the nanoparticles.

**FIGURE 5:**
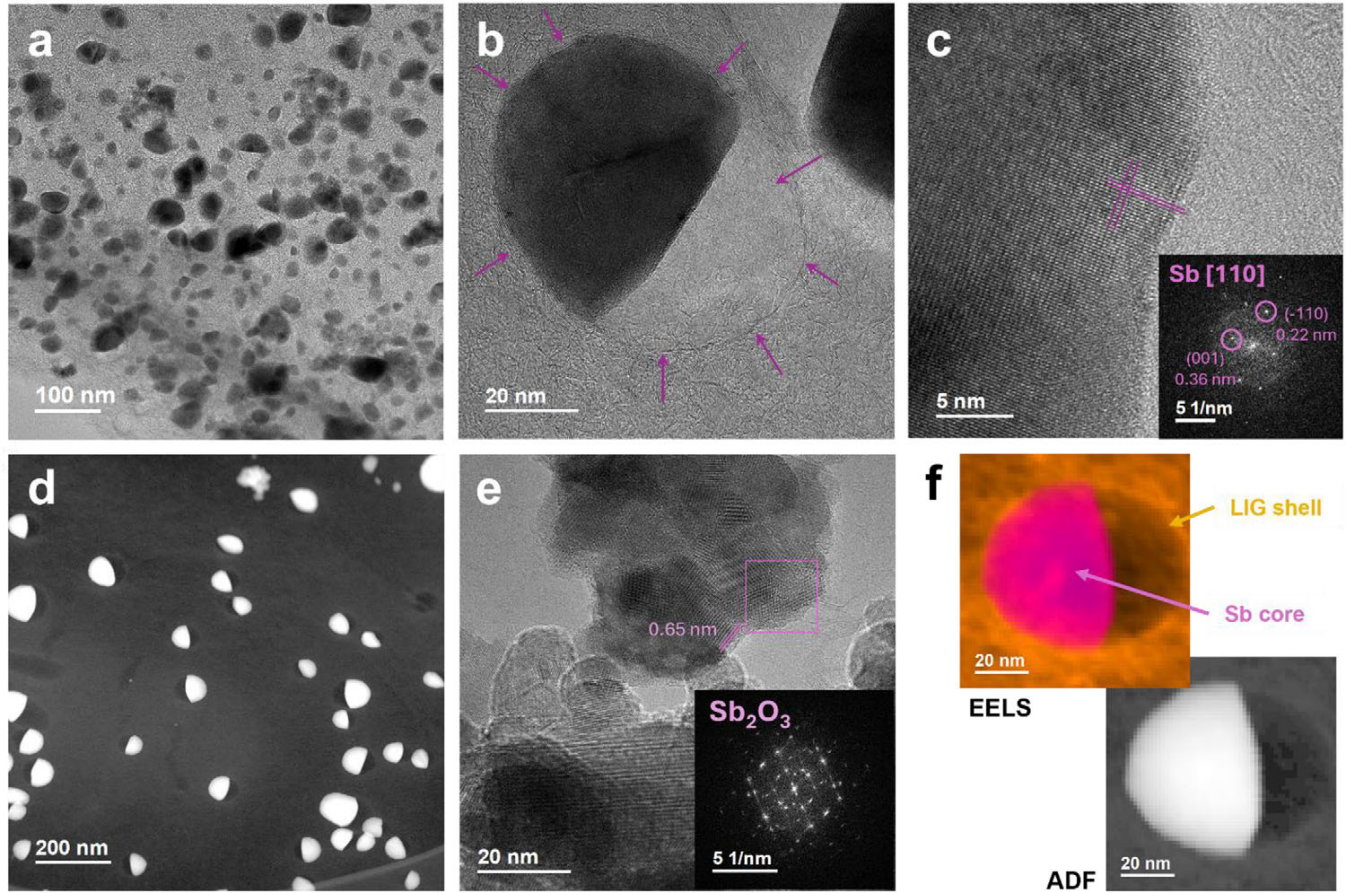
TEM analysis of LIG‐Sb sample. In figure (a), the HRTEM image shows the Sb‐NPS into the graphene matrix. Most of the particles evidence a taco‐like shape. (b) focusing on a Sb‐particle this shape is more evident, where the metal is confined in half of a carbon bubble, whose thickness consists of 3–5 graphene layers. The growth direction of Sb along (110) is reported in (c). (d) HAADF image displays with high contrast the Sb‐NPs shape and in the darkest color the empty space into the bubble. (e) Some NPs, concentrated in isolated bunches, belong to the Sb_2_O_3_ phase. (f) the EELS map of the Sb particle proves the formation of a coating layer of carbon from LIG containing the metallic Sb(0). Different from LIG‐Sn, no traces of core–shell structure are observed in this case.

A slightly different morphology was observed for the LIG‐Sb sample. Even in this case, most of the particles turn out to be metallic. However, the mean dimension resulted to be bigger than Sn‐NPs in LIG‐Sn. Indeed, a hierarchical distribution of particles is observed, but most of them turned out to be in the range of 20–50 nm (Figure 5a). Those particles present a taco‐like shape, surrounded by a circular cage made of a few graphene layers, corresponding to 3–5 in number. It appears like the Sb‐NPs are closed in a bubble (Figure 5b), and this peculiar shape is more evident in HAADF images, where the circular carbon bubble is half‐filled with Sb and half‐empty (Figure 5d). The peculiar shape seems to describe a previous formation of the graphene layers and consequently, a restriction of the active material inside. We hypothesized that the high‐temperature reached locally by the laser allows for the melting of the antimony, occurring at 630°C, on which the carbon cage is free to grow up. When the temperature is decreased, the solidification of Sb creates this taco‐shape. The edge of the particle displays the (110) facets on the flat side of the taco as shown in Figure 5c, and any traces of oxidate layer are observed by EELS map in Figure 5f. However, Sb_2_O_3_ is detected in bunches of isolated particles. The lower solubility of Sb(CH_3_CO_2_)_3_ likely leads to the formation of larger salt crystals, which decompose but are either not reduced or only partially reduced by the carbon matrix. These findings align with PXRD results and may explain the presence of a small percentage of the Sb_2_O_3_ phase. However, the presence of Sb_2_O_3_ is not considered critical for the performance of LIG–Sb, since the oxidized form of antimony can still act as an active material through the mechanism described in Equation (5). The main drawback is the irreversibility introduced during the first cycle, due to the formation of Li_2_O.

(5) $$\mathrm{Li}+Sb_{2}O_{3}\to Li_{2}O+Li_{3}\mathrm{Sb}$$

Operating in STEM mode, the EELS map was built to analyze the Sb‐NP, revealing the absence of a core–shell structure as observed in LIG‐Sn, but still evidencing the presence of a carbon cage protecting the particles from oxidation and pulverization upon cycling.

**FIGURE 6:**
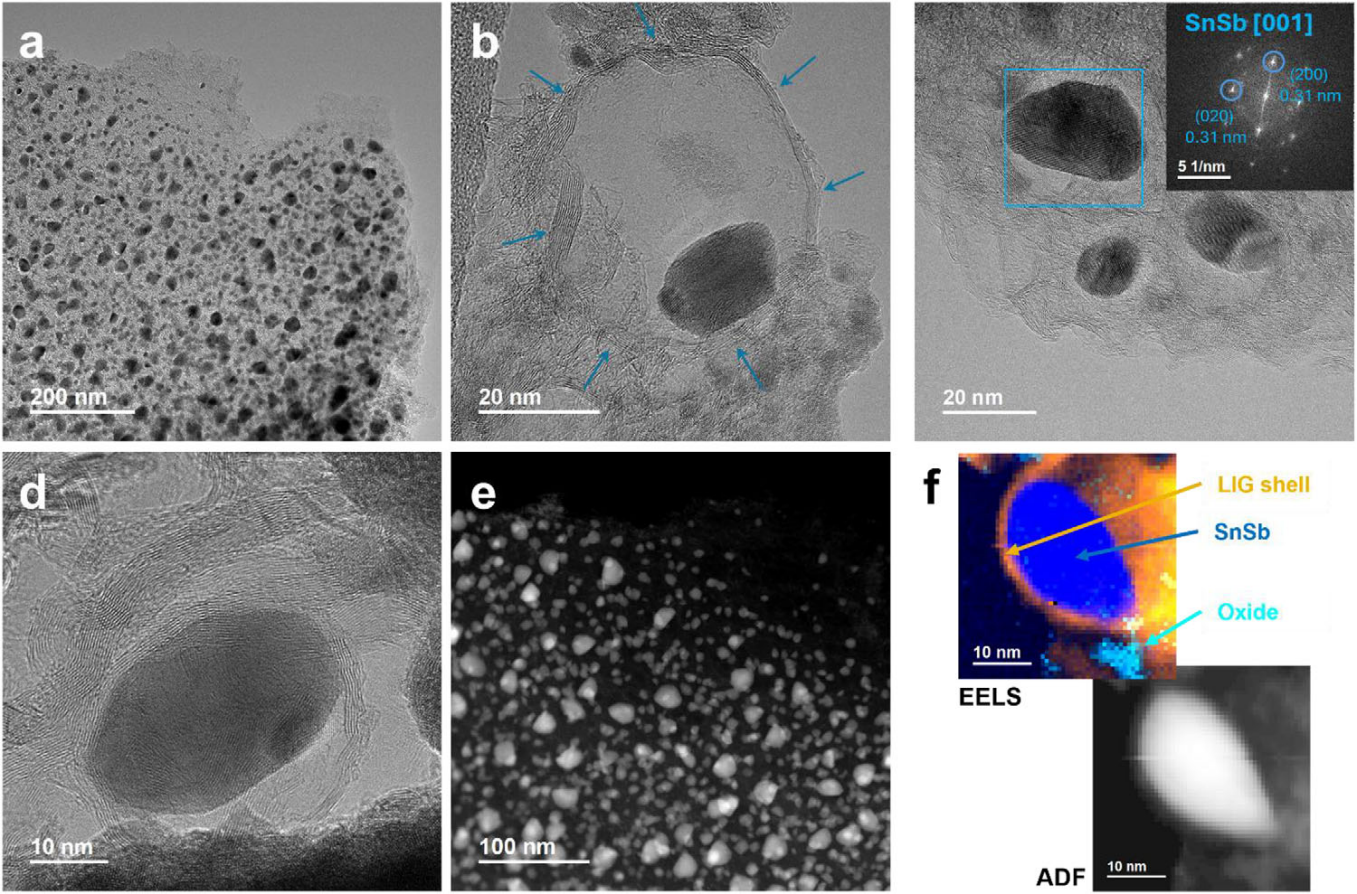
TEM analysis of LIG‐SnSb sample. In figure (a) it is evidenced the hierarchical distribution of particles in the graphene matrix. In (b,c) the SnSb NPs surrounded by a few graphene layers are displayed. The particles are encapsulated in a carbon bubble, with the presence of empty spaces around the particles. The SnSb phase is confirmed by the FFT of the particle. Smallest particles are observed at the edges, which can be attributed to the Sn(0) phase, which can form such small structures. In figure (d) a particle of Sb(0) is reported, confirming the presence of this phase in the matrix. HAADF is shown in (e), evidencing the taco‐shape of the NPs, while the EELS map in figure (f) clearly demonstrates the presence of SnSb particles surrounded by a carbon layer from LIG, with the presence of an empty space in the bubble.

The LIG‐SnSb sample exhibited a distinct morphology compared to LIG‐Sn, characterized by a lower degree of oxidation of the NPs, as already observed for LIG‐Sb. TEM analysis revealed that most of the nanoparticles were in the nanometer range, with sizes frequently below 10–20 nm (Figure 6a), encapsulated within a carbonaceous matrix. Unlike LIG‐Sn, where ultrasmall particles (< 2 nm) were abundant, the SnSb crystallites observed in LIG‐SnSb were significantly larger. No nanoparticles smaller than 2 nm ascribed to the SnSb phase were detected in this sample. Moreover, as observed previously. Most of the NPs present a taco‐shape like, encapsulated in a bubble of graphene, larger than the particle size (Figure 6b,e).

In addition to SnSb particles, smaller Sn‐NPs and larger Sb‐NPs were also identified, consistent with PXRD results that revealed a minor fraction of metallic antimony phases (Figure 6d). Despite the precursor salts being mixed in the correct molar ratio, as verified by EDX mapping, residual Sn atoms are likely to form sub‐nanometric particles undetectable by diffraction methods. EELS mapping of the SnSb nanoparticles indicated minimal oxygen content, with no evidence of surface oxidation layers (Figure 6f). Particularly suggests that the synthesis conditions, remarkably, the carbothermal reduction driven by the laser, effectively suppressed oxidation in the SnSb particles. The graphene matrix, formed through laser‐induced carbonization, displayed a stacked structure consisting of 6–8 layers, providing a robust template for nanoparticle embedding. This layered arrangement likely enhances the stability and conductivity of the composite, facilitating its application as an advanced anode material for Li‐ion and Na‐ion batteries.

**FIGURE 7:**
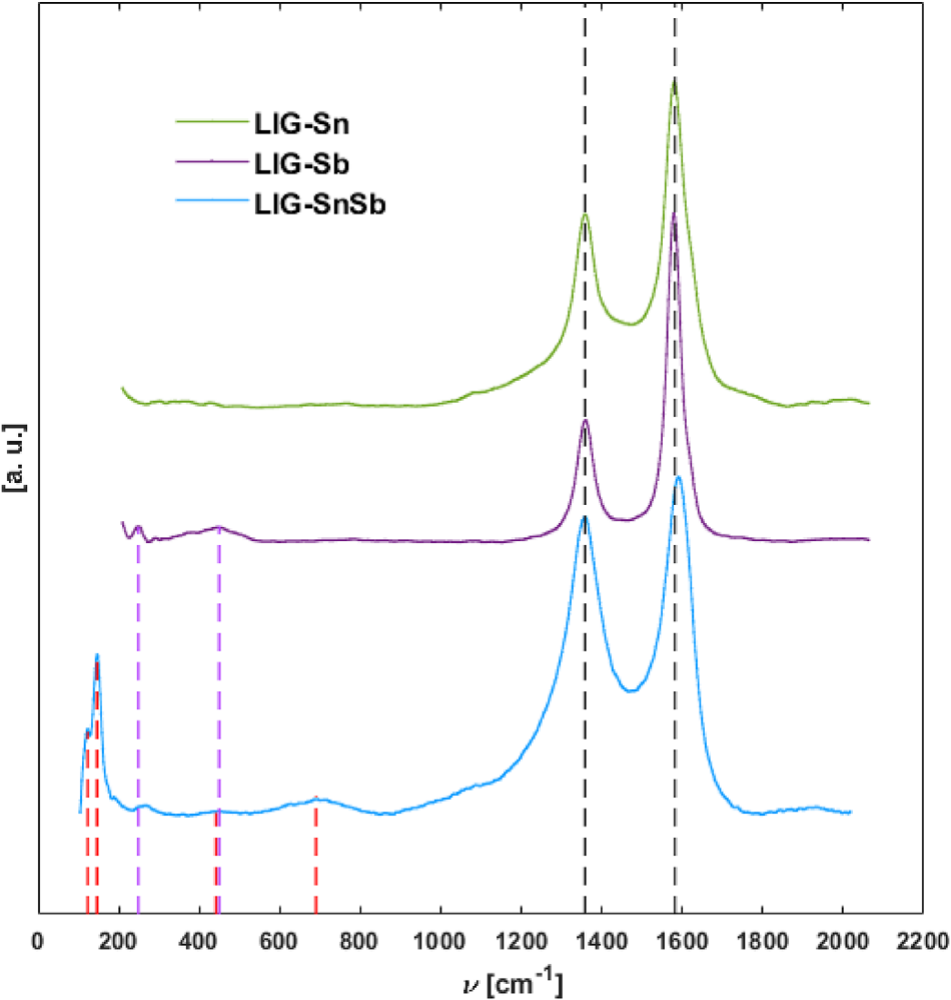
Raman spectra of LIG‐Sn, LIG‐Sb, and LIG‐SnSb are reported. Characteristic D and G bands at higher wavenumbers are reported, confirming the good quality of sp2 carbon in the matrix. Characteristic peaks of Sb2O3 and SnSb are marked [44].

Raman spectroscopy in Figure 7 confirmed the formation of graphene in all LIG‐metal composites, as evidenced by the characteristic D (1360 cm^−^^1^) and G (1580 cm^−^^1^) bands, which are typical of sp^2^‐hybridized carbon materials [41]. Notably, only the LIG‐SnSb sample exhibited a slight shift of the G band to 1589 cm^−^^1^.

At higher Raman shifts (see Figure S7), a single, broad 2D band was observed, indicating the presence of stacked graphene layers, as corroborated by TEM analysis. The Lorentzian shape of the 2D peak at 2717 cm^−^^1^ further confirms the formation of graphene, distinguishing it from the more structured 2D band typical of bulk graphite [41]. Although all three samples exhibited clear graphene signatures, the D/G intensity ratio varied slightly, reflecting differences in defect density and structural disorder across the samples.

For the LIG‐Sn sample, no discernible Raman signals corresponding to residual metal precursors or oxidation products have been detected, including SnO_2_, which was previously observed with TEM. This suggests that the thin oxide layer on Sn nanoparticles is either below the detection limit of Raman spectroscopy or its weak signal is masked by the carbon matrix.

In contrast, the LIG‐Sb spectrum was dominated by graphene features, and an additional peak at about 250 cm^−^^1^ and a broad band at about 450 cm^−^^1^ were detected, which is attributed to the Sb_2_O_3_ phase [38, 42], consistent with PXRD data.

The LIG‐SnSb sample displayed both graphene‐related features at high wavenumbers and peaks that could be compatible with SnSb alloy. Specifically, peaks at 123 and 146 cm^−^^1^ were assigned to Stistaite (SnSb), along with a broad spectral feature between 440 and 690 cm^−^^1^, confirming the successful formation of the SnSb phase [43, 44].

### 4.2 Electrochemical Measurements

To evaluate their potential for energy storage applications, electrodes based on LIG‐Sn, LIG‐Sb, and LIG‐SnSb were tested in Li‐ion and Na‐ion half‐cell configurations. Given their compatibility with both LIBs and SIBs, their electrochemical properties were systematically investigated.

**FIGURE 8:**
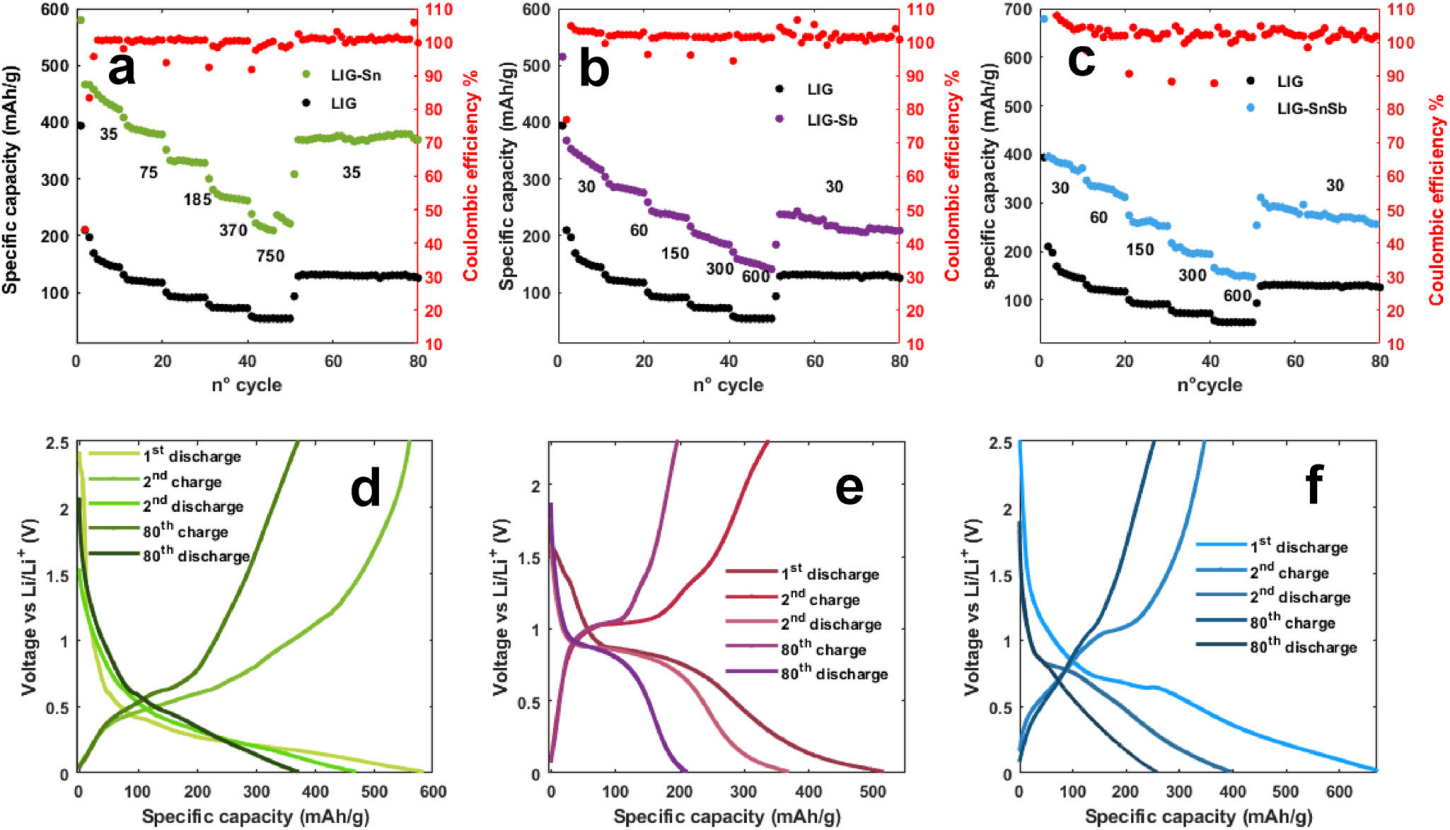
Discharge capacity over 80 cycles of (a) LIG‐Sn (mass loading 1.19 mg/cm^2^), (b) LIG‐Sb (mass loading 0.98 mg/cm^2^), and (c) LIG‐SnSb (mass loading 1.01 mg/cm^2^) compared to pure LIG electrodes. The numbers in the figure correspond to the current density (mA/g) applied to the electrode. GCD curves of the first, second, and 80th cycle are reported in (d) for LIG‐Sn, (e) for LIG‐Sb, and (f) for LIG‐SnSb. LIG‐Sn displays the typical curve of a tin‐based anode, with a large pseudo‐plateau below 0.5 V, LIG‐Sb displays the classical plateau around 0.9 V, while LIG‐SnSb displays a hybrid behavior.

In a Li‐ion half‐cell, the LIG‐Sn electrode was cycled between 0.01 and 2.5 V at a current density of 35 mA g^−1^. The electrode exhibited an initial discharge capacity of 579 mAh g^−1^, which dropped to 466 mAh g^−1^ in the second cycle before stabilizing at 378 mAh g^−1^ (Figure 8a,d). This initial capacity loss is primarily attributed to the solid electrolyte interphase (SEI) formation, a well‐known process involving irreversible side reactions that reduce the available lithium storage sites, while forming a protecting layer onto the electrode surface. As expected for Sn‐based anodes, the discharge profile featured characteristic plateaus below 0.5 V vs. Li/Li^+^ as reported in Figure 8d, corresponding to the stepwise alloying reaction of Sn with Li to form Li_x_Sn intermetallic phases [11, 45]. The charge process exhibited plateaus at 0.4 and 0.6 V vs. Li/Li^+^, indicative of the dealloying process. Despite the initial capacity fading, the electrode demonstrated excellent cycling stability, with Coulombic efficiency consistently approaching 100% and no significant capacity degradation even after 80 cycles (Figure 8a). A significant enhancement in capacity was observed when comparing LIG‐Sn to pure LIG, with the Sn‐decorated electrode achieving a capacity three times higher than pure LIG. Furthermore, the presence of Sn in the form of NPs, combined with the conductive thin graphene layers shell, facilitated the diffusion of ions in the structure, even at higher current rates, allowing superior rate capability. At the current density of 750 mA g^−1^, the electrode retained a specific capacity of 225 mAh g^−1^, underscoring its potential for high‐power applications.

Under identical cycling conditions, the LIG‐Sb electrode exhibited the characteristic behavior of Sb‐based anodes, with a distinct plateau at 0.9 V vs. Li/Li^+^ during discharge (Figure 8e), corresponding to the formation of Li_3_Sb [17, 21]. The initial capacity was 515 mAh g^−1^, which dropped to 367 mAh g^−1^ in the second cycle and continued to decrease over subsequent cycles as reported in Figure 8b. Unlike LIG‐Sn‐based electrodes, LIG‐Sb follows a single‐step lithiation process, as reflected by the absence of multiple plateaus in the galvanostatic profiles. During charging, the electrode exhibited the expected delithiation plateau at 1.1 V vs. Li/Li^+^, characteristic of Li_3_Sb decomposition. However, if compared to LIG‐Sn, the capacity retention was significantly lower, with a steady decline over cycling. Despite Coulombic efficiency remaining close to 100%, the progressive capacity loss is consistent with literature reports highlighting the higher structural instability of Sb compared to Sn in lithium‐ion systems [46]. Nevertheless, the graphene matrix in LIG‐Sb improved electrode durability as compared to pure Sb anodes, which typically suffer from rapid capacity decay. While pure Sb electrodes fail within a few cycles, the LIG‐Sb electrodes remained operational even after 80 cycles, stabilizing at a specific capacity of ∼210 mAh g^−1^, performing better than pure LIG electrodes.

The LIG‐SnSb electrode exhibited hybrid electrochemical behavior, incorporating features of both Sn and Sb‐based electrodes. The discharge profile displayed a small plateau at 0.9 V vs. Li/Li^+^, corresponding to Li_3_Sb formation, while a broader slope below 0.5 V indicated the formation of Li_x_Sn (0 < x < 4.4) [47, 48] intermetallic phases (Figure 8f). A significant initial capacity fade was observed, with the discharge capacity decreasing from 679 mAh g^−1^ in the first cycle to 395 mAh g^−1^ in the second cycle, due to SEI formation and possible degradation of larger particles (see Figure 8c). However, following this early loss, the electrode exhibited remarkable cycling stability, outperforming both LIG‐Sn and LIG‐Sb over extended cycling. The specific capacity stabilized at ∼256 mAh g^−1^, benefiting from the reversible alloying reactions of SnSb. The superior capacity retention of LIG‐SnSb is attributed to the peculiar graphene‐based carbon matrix arranged in the taco‐shape observed through TEM images, which acts as a mechanical buffer and volume containing of the particle, preventing electrode pulverization and enhancing structural integrity. Notably, when comparing the third and 80th cycles, the capacity and voltage profiles remained nearly identical, confirming the long‐term stability of the SnSb alloy. At increased current densities, the electrode maintained a high‐rate capability, with a specific capacity of ∼150 mAh g^−1^ at 600 mA g^−1^, further demonstrating its suitability for high‐power applications. All reported capacities account for the total electrode mass on the collector.

Considering only the metal active material (Sn, Sb, and SnSb) in the electrode, their specific capacities were calculated. The specific capacities of pure LIG were measured at different current rates and are reported in Figures S5 and S6. EDX measurements revealed that the metal ratio is almost 50% by mass, allowing the specific capacity of the carbon component to be accounted for and the values for the metallic active mass component to be extracted. Based on this, the specific capacities were determined as follows: tin in LIG‐Sn 624 mAh g^−1^, antimony in LIG‐Sb 305 mAh g^−1^, and the SnSb alloy in LIG‐SnSb 465 mAh g^−1^.

A comparison of the obtained results with literature data confirms the effective integration of metallic particles within the composite electrodes. This simple synthetic approach enables the formation of a protective carbon shell, which mitigates particle degradation during charge–discharge cycles, thereby enhancing electrochemical performance. Table 1 presents a direct comparison between literature reports and the results achieved in this work.

**TABLE 1:** Comparison of this work with literature results for Sn, Sb, and SnSb‐based LIB anode.

	Preparation	Specific Capacity (mAh g^−1^)	Refs.
SnO nanoplate	Spray pyrolysis	330 (0.10 A g^−1^)	[49]
SnO_2_ NPs anchored on graphene	Hydrothermal method	430 (0.10 A g^−1^)	[50]
Sn‐NPs anchored on carbon nanofiber	Hydrothermal method and electrospinning	460 (0.2 A g^−1^)	[13]
Sn‐NPs encapsulated in hollow carbon sphere	Template method and carbonization	550 (0.17 A g^−1^)	[11]
Graphene‐coated Sn‐NPs anchored on 3D porous graphene network	Template‐assisted CVD	1089 (0.2 A g^−1^)	[51]
LIG‐Sn	Laser assisted carbonization	624 (0.07 A g^−1^)	This work
Sb‐carbon nanocomposite	Ball‐milling	400	[52]
Sb embedded in carbon nanosheets	Thermo‐chemical reduction	230	[53]
Three‐dimensional antimony nanochains	Amine‐boranes metal reduction	430	[18]
Antimony‐carbon nanosponge	Citrate gel synthesis	153	[17]
Sb/rGO	Binder‐free Electrophoretic	170	[54]
LIG‐Sb	Laser assisted carbonization	305 (0.06 A g^−1^)	This work
SnSb NCs	Solution chemical reaction	>700 (0.35 A g^−1^)	[48]
SnSb NP sinto hollow carbon nanofiber	Electrospinning and carbonization	646 (0.05 A g^−1^)	[55]
Tin antimony oxide @graphene	Hydrothermal	648 (0.5 A g^−1^)	[56]
Nano‐SnSb deposited on MCMB	Co‐precipitation	420	[57]
LIG‐SnSb	Laser assisted carbonization	465 (0.06 A g^−1^)	This work

Notably, this straightforward synthesis method simultaneously facilitates the formation of metallic Sn, Sb, and SnSb nanoparticles embedded within graphene layers, eliminating the need of complex chemical pathways or high‐temperature treatments. Compared to most literature reports, the active elements in this system exhibit remarkable stability, retaining functionality even after 80 cycles. These findings highlight this novel approach as a promising alternative for the scalable synthesis of carbon‐metal composites for LIB anode applications. After cycling, all the half‐cells were opened. Good adhesion of the active material to the copper collector, with no sign of delamination, was observed.

The same electrodes were subsequently tested in a half‐cell configuration against sodium, assembled as described in the previous section. The voltage window was set between 2.5 and 0.01 V vs Na/Na+, with an initial current density of 10 mA g^−1^, which was later increased to evaluate performance at higher charge–discharge rates.

**FIGURE 9:**
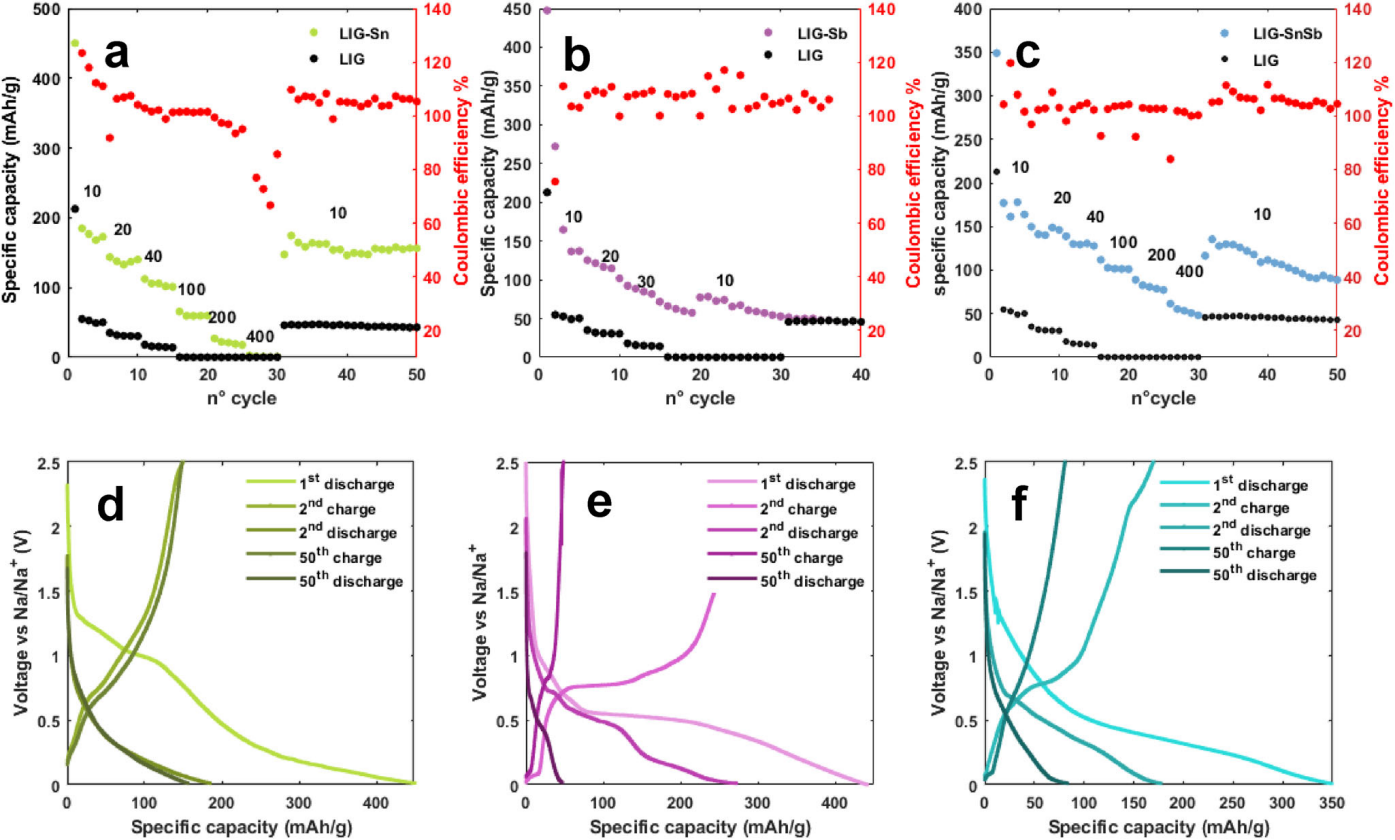
Discharge capacity over 50 cycles of (a) LIG‐Sn, (b) LIG‐Sb and (c) LIG‐SnSb in SIB half‐cell. The numbers in the figure correspond to the current density (mA/g) applied to the electrode. GCD curves of the first, second, and 80th cycle are reported in (d) for LIG‐Sn, (e) for LIG‐Sb, and (f) for LIG‐SnSb. LIG‐Sn display the typical curve of tin‐based anode, with a large pseudo‐plateau below 0.5 V vs Na/Na^+^, hence LIG‐Sb displays the classical plateau around 0.7 V vs Na/Na^+^ while LIG‐SnSb combines characteristics of both the other individual electrodes.

The LIG‐Sn electrode exhibited an initial discharge capacity of 446 mAh g^−1^, which rapidly decreased to 155 mAh g^−1^, stabilizing at this value after 50 cycles. The significant first‐cycle irreversibility is mainly attributed to SEI formation and electrode‐electrolyte interactions, as well as the disintegration of larger particles within the matrix. The complete sodiation of tin (Na_4_Sn) results in an extreme volume expansion of ∼420%, significantly higher than that observed in lithium cells, leading to potential electrode pulverization [15, 58]. However, after this initial capacity loss, the electrode maintains stable performance. The GCD curves (Figure 9d) clearly illustrate the role of Sn in the electrode, with pseudo‐plateaus appearing below 0.5 V vs Na/Na^+^ during discharge, corresponding to multiple sodiation steps, while charge plateaus extend up to 0.63 V vs Na/Na^+^. The coulombic efficiency remains consistently close to 100%, highlighting the high reversibility of the system (Figure 9a). At higher current rates, capacity decreases rapidly, probably due to the slow diffusion kinetics of sodium, but it recovers to its original value once the current is replaced to its initial value.

In contrast, the LIG‐Sb electrode, similar to what is observed in LIBs, exhibits poor stability against sodium. The first‐cycle capacity reaches approximately 450 mAh g^−1^, but it rapidly declines to 135 mAh g^−1^after just four cycles. At higher current rates, the electrode experiences severe capacity loss and fails to operate above 40 mA g^−1^. The voltage profile of LIG‐Sb (Figure 9e) shows a characteristic pseudo‐plateau at 0.56 V vs Na/Na^+^ during discharge, corresponding to Sb sodiation, while the plateau at 0.74 V vs Na/Na^+^ during charge indicates desodiation [59, 60, 61]. The instability of this electrode is primarily attributed to the large particle size of antimony, which promotes pulverization and structural degradation due to its ∼300% volume expansion upon sodiation.

The LIG‐SnSb electrodes demonstrate improved stability, displaying an intermediate behavior between LIG‐Sn and LIG‐Sb. The initial capacity reaches 350 mAh g^−1^, quickly stabilizing at 160 mAh g^−1^. Compared to LIG‐Sb, the SnSb electrodes exhibit better cycling performance, retaining nonzero capacity even at high current densities. Specifically, at 400 mA g^−1^, the specific capacity remains at ∼50 mAh g^−1^ (Figure 9c). However, LIG‐Sb stability upon cycling is lower to that of LIG‐Sn, as the capacity gradually declines, resulting in poor retention over extended cycling. The discharge profile highlights a pseudo‐plateau below 0.7 V vs Na/Na^+^, associated with the sodiation of antimony in the SnSb alloy, followed by a second plateau below 0.5 V vs Na/Na^+^, attributed to the multiple sodiation reactions of tin [19].

Comparing the obtained results with the literature, the LIG composite exhibits lower specific capacity values, indicating the need for further improvements. However, despite the lower capacities, the findings highlight the potential of LIG to enhance the cyclability of Sn, Sb, and SnSb as anode materials in SIBs by providing a protective cage around the particles (Table 2).

**TABLE 2:** Comparison of this work with literature results for Sn, Sb and SnSb‐based SIB anode.

	Preparation	Specific Capacity (mAh g^−1^)	Refs.
Ni_x_Sn	Colloydal synthesis	160 (0.1 A g^−1^)	[62]
Sn nanowall‐shaped arrays	Electrodeposition	801 (0.16 A g^−1^)	[63]
Sn nanodots embedded inside N‐doped microcages	Pyrolysis and calcination	332	[64]
Encapsulated SnO_2_ NPs into holey CNTs	Hydrothermal	234 (0.1 A g^−1^)	[65]
LIG‐Sn	Laser assisted carbonization	250 (0.02 A g^−1^)	This work
Monodisperse Sb nanocrystals	Colloidal synthesis	500 (0.33 A g^−1^)	[66]
Porous antimonene	Electrochemical exfoliation	570 (0.1 A g^−1^)	[67]
Sb@TiO_2‐x_ nanoplates	Salt‐template method	568 (0.1 A g^−1^)	[68]
Sb/N‐doped layered carbon	Coprecipitation and annealing	280 (1 A g^−1^)	[69]
LIG‐Sb	Laser assisted carbonization	50 (0.02 A g^−1^)	This work
SnSb NCs	Solution chemical reduction	350 (0.66 A g^−1^)	[48]
3D SnSb@N‐PG	Spray drying method	190 (10 A g^−1^)	[70]
Fe_0.1_‐SnSb alloy	Rapid solidification	500 (0.05 A g^−1^)	[71]
3D SnSb@SnO_x_/SbO_x_@C	NaCl template assisted in situ catalytic method	195 (2 A g^−1^)	[72]
LIG‐SnSb	Laser assisted carbonization	150 (0.02 A g^−1^)	This work

## 5 Conclusions

In this study, the thermo‐conversion induced by laser of PAA precursor enriched with metal salt precursors was investigated; this process leads to the simultaneous graphitization of a plastic substrate and its functionalization with metallic NPs of Sn, Sb, and the SnSb alloy, thanks to the carbothermal reduction of the metal sources. This facile, one‐step approach enables the direct synthesis of anode materials for both LIBs and SIBs, offering an efficient alternative to conventional, multi‐step electrode fabrication processes. Furthermore, this method facilitates the formation of Sn, Sb, and SnSb NPs embedded within a few layers of graphene coating, which not only anchors the active material to the electrode but also mitigates the volume expansion during lithiation and sodiation. LIG composites exhibited improved performance compared to bare LIG in both LIBs and NIBs, confirming the active role of metals in the electrodes. However, the synthetic approach used in this study does not allow separation of the metallic particles from the LIG matrix; therefore, the specific contribution of graphene cannot be directly evaluated. Nevertheless, the observed cycling stability strongly supports the role of graphene in enhancing the long‐term stability of the cells. Additionally, the direct synthesis of the active material on a copper current collector results in a binder‐free anode, enhancing its practical application.

Among the LIG composites considered in this work, LIG‐Sn demonstrated the best electrochemical performance, which was attributed to the ultrasmall size of the nanoparticles. The composite exhibited a stable specific capacity of 378 mAh g^−1^ after 80 cycles in LIBs and 155 mAh g^−1^ in SIBs. When considering only the mass of tin, the specific capacities reached 624 and 250 mAh g^−1^, respectively. In contrast, the LIG‐Sb composite displayed lower electrochemical performance and poor cycling stability, primarily due to the larger particle size of Sb within the matrix. The LIG‐SnSb composite exhibited intermediate electrochemical properties, showing improved specific capacity and cycle stability in both LIBs and SIBs compared to LIG‐Sb. However, its capacity retention remained lower than that of LIG‐Sn. After 80 cycles in LIBs, LIG‐SnSb achieved a specific capacity of 256 mAh g^−1^, while in SIBs, it reached 89 mAh g^−1^ after 50 cycles.

In conclusion, this simple and scalable synthetic method demonstrates the feasibility of fabricating high‐performance anode materials for LIBs and SIBs. While further optimization of synthesis parameters may yield even more promising results, this study establishes a proof‐of‐concept for the direct synthesis of LIG‐metal composites on a copper current collector. The laser‐induced carbothermal reduction not only graphitizes the PAA substrate but also facilitates the in situ reduction of metal salts, highlighting its potential as a viable alternative for next‐generation battery electrode production.

## Conflicts of Interest

The authors declare no conflicts of interest.

## Supporting information




**Supporting File**: gch270052‐sup‐0001‐SuppMat.docx.

## Data Availability

The data that support the findings of this study are openly available in [Zenodo] at [https://doi.org/10.5281/zenodo.15639524], reference number [15639524].
