# Supplementary material for: Simultaneous Laser Reduction of Sn/Sb Salts and Graphene Formation as Innovative Anode Material for Li‐ and Na‐Ion Batteries
Source: Glob Chall. 2026 Apr 29;10(5):e00356. doi: 10.1002/gch2.202500356 (PMC13128972; doi:10.1002/gch2.202500356)
Supplement: Supplementary file 1 — Supporting File: gch270052‐sup‐0001‐SuppMat.docx. [file GCH2-10-e00356-s001.docx]

SIMULTANEOUS LASER REDUCTION OF Sn/Sb SALTS AND GRAPHENE FORMATION AS INNOVATIVE ANODE MATERIAL FOR LI- AND NA-ION BATTERIES

*Vincenzo Vezzoni^ab^, Michele Setti^ab^, Giacomo Magnani^ab^, Laura Fornasini^c^, Silvio Scaravonati^ab^, Alessia Rinaldi^ab^, Niyaz Ahmad^ab^, Lorenzo Pasetti^c^, Danilo Bersani^c^, Giovanni Bertoni^d^, Michele Sidoli^ab^, Mauro Riccò^ab^, Daniele Pontiroli^ab^^[[1]](#footnote-1)^**

1. Nanocarbon Laboratory, cIDEA & Department of Mathematical, Physical and Computer Sciences, University of Parma, Parco Area delle Scienze 7/A, 43124, Parma, Italy
2. GISEL National Centre of Reference for Electrochemical Energy Storage Systems, INSTM National Interuniversity Consortium of Materials Science and Technology, Via Giusti 9, 50121 Firenze, Italy
3. Department of Mathematical, Physical and Computer Sciences, University of Parma, Parco Area delle Scienze 7/A, 43124 Parma, Italy
4. CNR – Istituto Nanoscienze, Via Campi 213/A, 41125, Modena, Italy

## OPTIMIZATION OF LASER PARAMETERS

For the optimization of laser parameters, the electrical conductivity between the upper part of the electrode and the copper collector was considered. To evaluate the best parameters, the resistance has been measured using a multimeter between the black part and the copper collector. Firstly, the focus depth was optimized, varying between 39 mm to 44 mm of distance between the laser source and the PAA substrate. The best result, shown in Figure S1, displays an optimal contact for Z=42 mm. After that, the power and speed of the laser were tuned to reduce the resistance, resulting in best setting of 1440 mW of power and 500 mm/min of speed. The resistance between the active material (LIG) and the current collector can be additionally decreased with a second passage of the laser, allowing for contact-resistance <20 Ω.


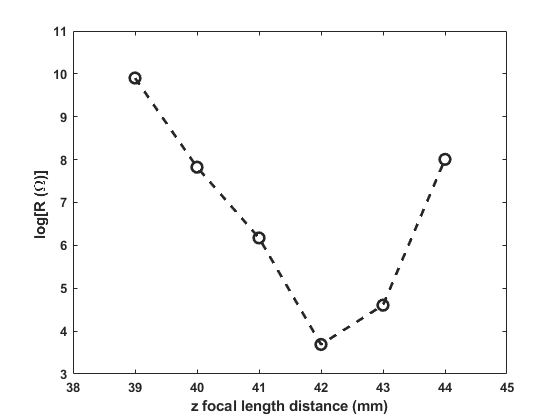


Figure S1: Logarithm of the resistance between the active material (LIG) and the copper current collector is reported as a function of the focal length (z) between the laser and the substrate. This parameter is crucial for the good conversion of PAA substrate, which ensure a proper electric contact between the active material and the current collector.

## PXRD


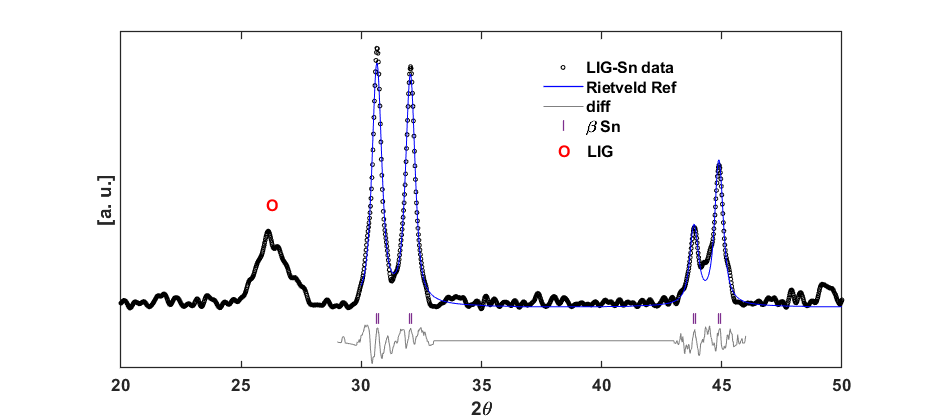


Figure S2 PXRD pattern of LIG-Sn and the corresponding Rietveld refinement (Rwp = 9%). The peaks are associated to the β-Sn structure of metallic Sn, while the large band at 2θ=26° is ascribed to LIG contribution.


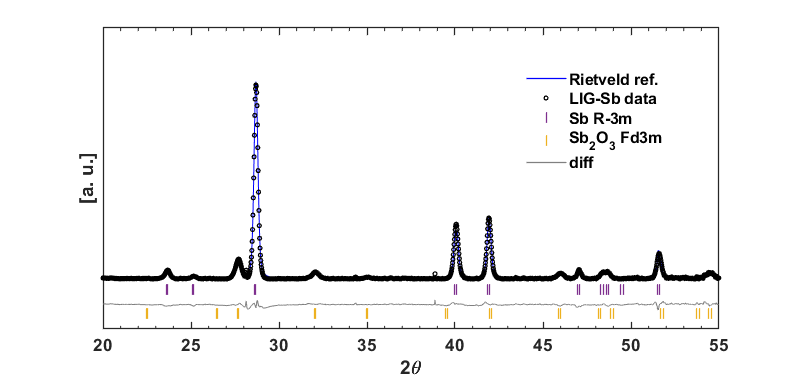


Figure S3 PXRD pattern of LIG-Sb and the corresponding Rietveld refinement (Rwp = 5.5%). The peaks indicized in purple correspond to the rhombohedral structure of metallic Sb, while yellow tickmarks are associated to the Sb_2_O_3_ cubic phase. The resulting phase fraction turn out to be 12% of Sb_2_O_3_ and 88% of metallic Sb.


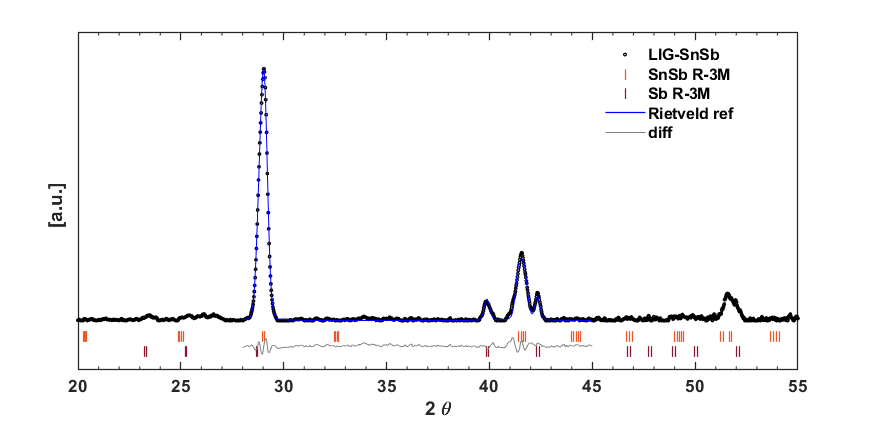


Figure S4 PXRD pattern of LIG-SnSb and the corresponding Rietveld refinement (Rwp = 4.6%). The main peaks are associated to the stistaite phase structure, while the other peaks are meanly related to the metallic Sb phase which resulted to be 9% in mass from the QPA analysis.

## SEM ANALYSIS


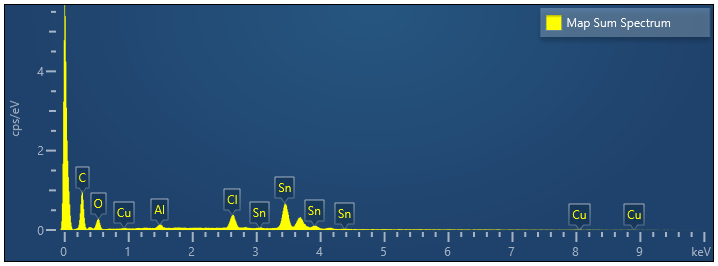


| **Map Sum Spectrum** |  |  |  |  |
| --- | --- | --- | --- | --- |
| **Element** | **Line Type** | **Weight %** | **Weight % Sigma** | **Atomic %** |
| C | K series | 31.28 | 0.46 | 68.51 |
| O | K series | 8.82 | 0.29 | 14.50 |
| Al | K series | 0.95 | 0.07 | 0.93 |
| Cl | K series | 5.61 | 0.15 | 4.16 |
| Cu | L series | 0.40 | 0.17 | 0.17 |
| Sn | L series | 52.94 | 0.50 | 11.73 |
| Total |  | 100.00 |  | 100.00 |

Figure S5: EDX spectrum from SEM analysis of LIG-Sn sample. The elemental analysis evidence the presence of chloride, suggesting that not all the SnCl_2_ inside the sample is completely decomposed.


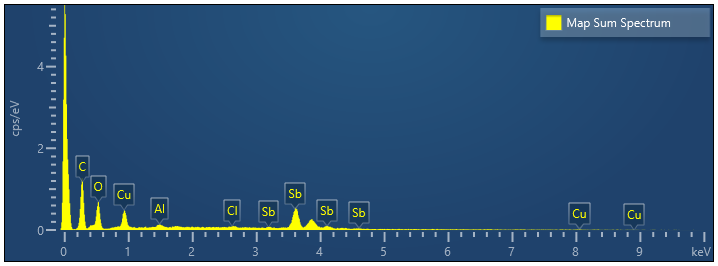


| **Map Sum Spectrum** |  |  |  |  |
| --- | --- | --- | --- | --- |
| **Element** | **Line Type** | **Weight %** | **Weight % Sigma** | **Atomic %** |
| C | K series | 33.29 | 0.61 | 69.82 |
| O | K series | 9.79 | 0.30 | 15.42 |
| Al | K series | 0.65 | 0.11 | 0.61 |
| Cl | K series | 0.58 | 0.13 | 0.41 |
| Cu | L series | 11.66 | 0.42 | 4.62 |
| Sb | L series | 44.03 | 0.67 | 9.11 |
| Total |  | 100.00 |  | 100.00 |

Figure S6: EDX spectrum of LIG-Sb. Considering the Sb percentage excluding spurious atoms such as copper, the percentage of Sb is close to 50 % in mass.

## RAMAN SPECTROSCOPY

The Raman spectrum at higher Raman shift, acquired as described in the main text, shows the typical form of graphene-like structure with a single band at 2717 cm^-1^ with FWHM of 42±1 cm^-1^ fitted by a single Lorentzian peak. This shape confirms the formation of graphene, since the spectrum is highly different compared to graphite Raman spectrum, which displays a more structured peak in this region, coming from multiple contributions^1^.


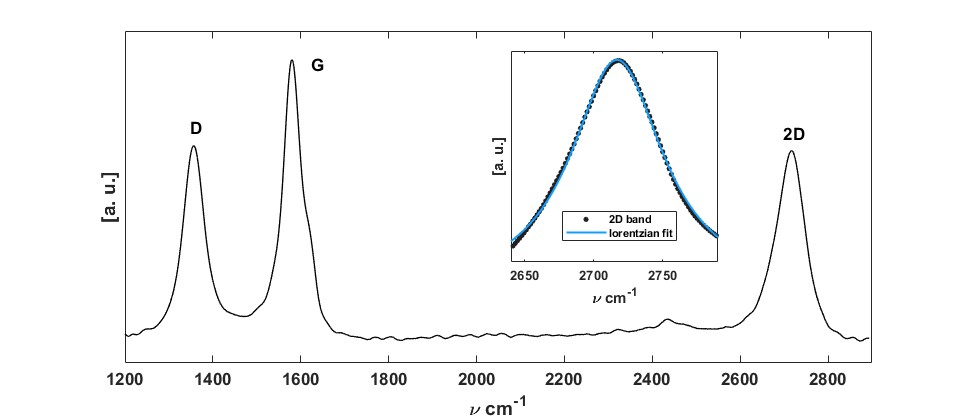


Figure S7 Raman spectrum of LIG-Sn displays the characteristic graphene D and G band at 1355 cm^-1^ and 1580 cm^-1^ respectively. At higher Raman shift the presence of the 2D band is reported at 2717 cm^-1^. In the inset figure the Lorentzian fit of the 2D band is reported confirming the graphemic origin of the signal.

## LIG Li-ion and Na-ion HALF CELL

To establish a standard, the electrochemical properties of bare LIG were first investigated and compared to LIG modified with nanoparticles. During the charge and discharge process, Li ions can intercalate between the graphene planes due to its multilayer structure and can also fill the smaller pores. The LIG vs. metallic lithium half-cell was assembled as described in section 4.2.2, using a coin cell cycled between 0.01 V and 2.0 V (vs. Li/Li⁺), with a fixed current at various C-rates to evaluate the performance of LIG at different current densities. The current densities were chosen in the range between 30 mA g^-1^ and 600 mA g^-1^. Initially, the upper voltage limit was set at 2.5 V, but during the first two cycles, an undesirable plateau behavior above 2 V was observed, likely linked to unwanted side reactions. As a result, the upper limit was adjusted to 2.0 V. After each charge and discharge cycle, a 30-minute rest period was included to stabilize the cell’s potential and allow lithium ions to fully intercalate into the graphene structure.

The charge and discharge profiles, shown in *Figure S8a*, clearly display a high degree of irreversibility in the first cycle, which is consistent with the formation of the SEI at the interface between the anode material and the electrolyte. The large surface area of LIG likely promotes SEI formation, contributing to the high irreversibility after the first cycle. However, the charge-discharge profiles tend to stabilize after a few cycles, leading to a consistent specific capacity.

Unlike conventional graphite, graphene does not exhibit a flat plateau during cycling^2^. Instead, the voltage profile shows a slope-like curve as reported in *Figure S8b*, reflecting various Li-ion adsorption mechanisms on the surface and within the pores and stacked graphene layers. The reversible specific capacity of the LIG anode is approximately 115 mAh g^-1^ (*Figure S9*), which is lower than that of other graphene electrodes for LIBs, and significantly lower than the standard reference material, graphite. Literature reports higher specific capacities for LIG anodes^3^, reaching up to 500 mAh g^-1^. A possible explanation for the reduced capacity is the incomplete conversion of the polyimide substrate, leaving a significant amount of non-active material on the electrode surface.

The total electrode mass was used in the specific capacity calculation, and since this electrode is fabricated without a binder, it is likely that part of the material is not fully converted into LIG. This unconverted material can act as non-active mass, potentially distorting the specific capacity calculations; however, it is essential for assuring good adhesion of the active material to the current copper collector.


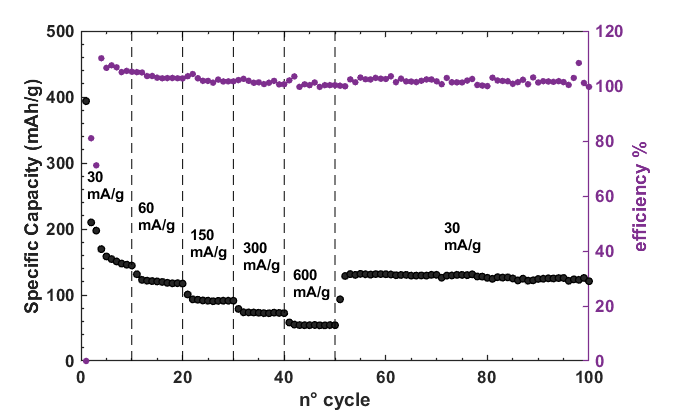


Figure S9 Specific discharging capacity of bare LIG are reported at different current rate, upon cycles. After few cycles the Specific Capacity is stabilized at constant values, displaying good capacity retention.


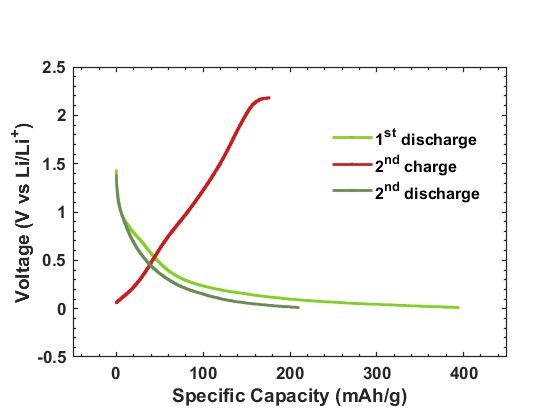

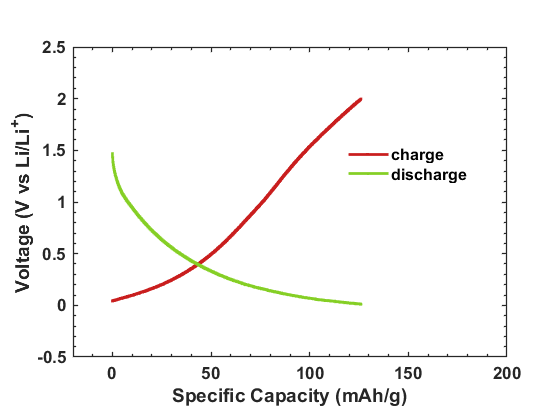


Figure S8 a) First and second cycle discharge profile of bare LIG electrode vs Li/Li^+^ (discharge rate of 30 mA g^-1^) is reported. During the charge process a pseudo-plateau at voltage > 2V is observed due to spurious reaction with the electrolyte. b) the charge-discharge profile after stabilization and limited at 2 V is reported, showing the characteristics slope-shape of graphene-based anode material.

**a)**

**b)**

LIG in sodium ion half-cell has been tested as well. The material has been cycled between 0.01V and 2.50 V vs Na/Na^+^, displaying a large specific capacity during the first discharge which quickly stabilizes at 45 mAh g^-1^. The scan rate was changed between 10 mA g^-1^ to 100 mA g^-1^, evidencing a great loss of capacity which decreased to zero at 100 mA g^-1^. However, the electrochemical properties are almost stable upon cycling, resulting in an almost constant value upon cycling.

**b)**

**a)**


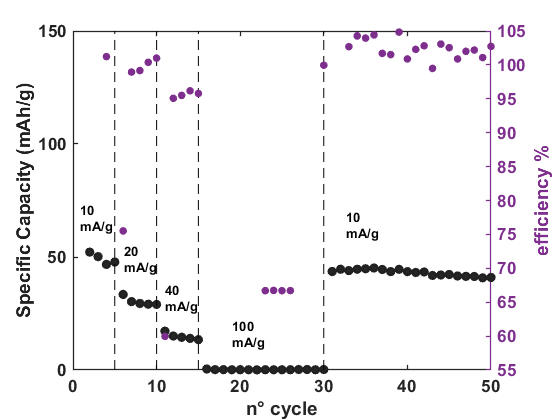


Figure S11 Specific discharging capacity of bare LIG in SIB half-cell are reported at different current rate, upon cycles. The capacity goes to zero at higher rate, but it becomes constant upon cycling with good capacity retention.


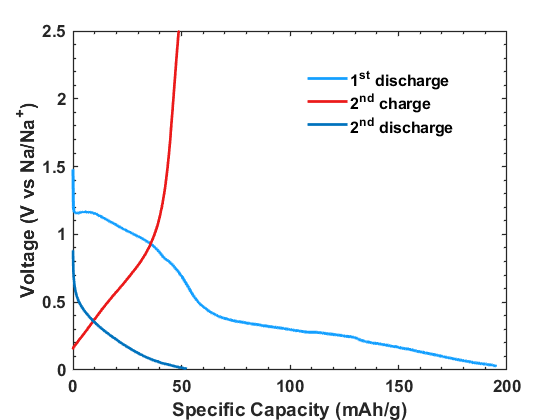

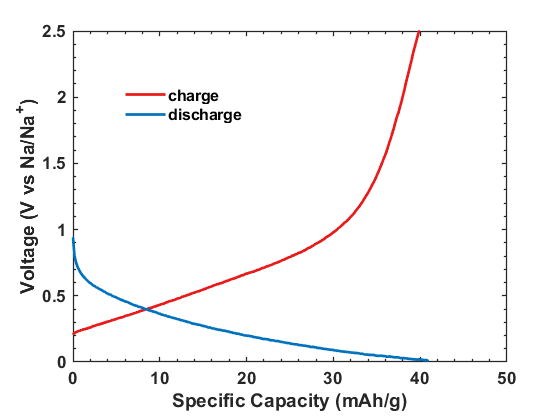


Figure S10 a) First and second cycle discharge profile of bare LIG electrode vs Na/Na^+^ (discharge rate of 10 mA g^-1^) is reported. b) the charge-discharge profile after stabilization and limited at 2.5 V is reported, showing the characteristics slope-shape of graphene-based anode material.

The discharge curve of LIG composite at different current rates in Li half-cell are reported. The composite LIG-Sn displays the best performance even at 750 mA g^-1^ which means a charging time of less than 30 minutes (*Figure S12a*). From the curve, the active role of tin into the composite is clear, due to the presence of a pseudo-plateau at potential lower than 0.5 V vs Li/Li^+^. LIG-Sb specific capacity quickly decreases at higher current rates; however, the active role of antimony is guaranteed by the characteristic plateau at voltage lower than 0.9 V vs Li/Li^+^ shown in *Figure S12b*. In *Figure S12c* the behavior of LIG-SnSb composite is reported. Even in this case the capacity is reduced at higher current rate, but still the role of SnSb is evidenced by the characteristic discharge profiles.


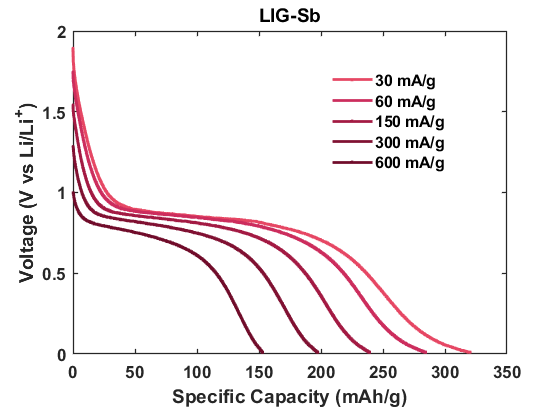

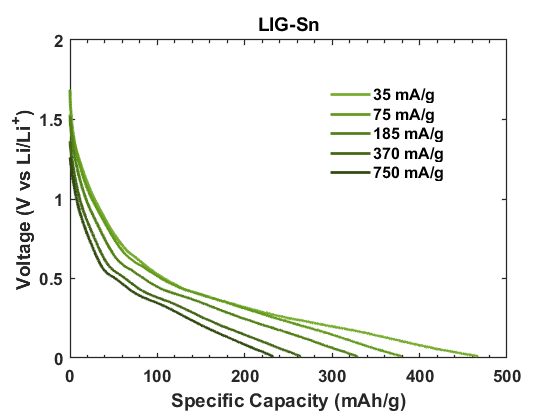

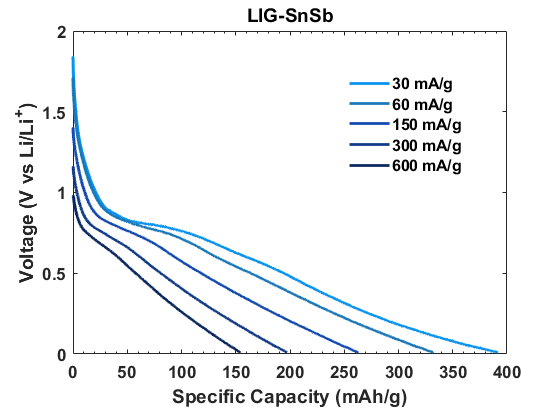

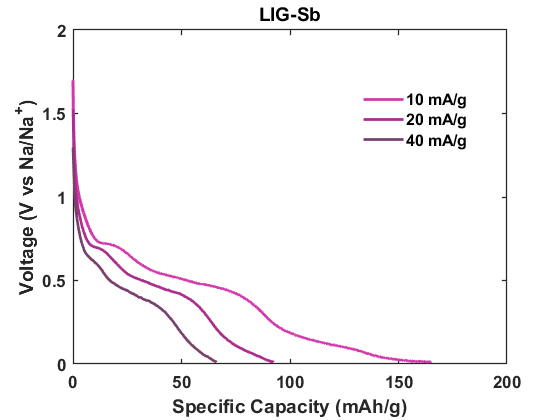

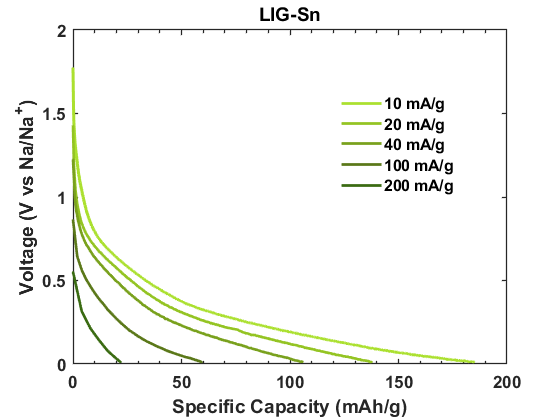

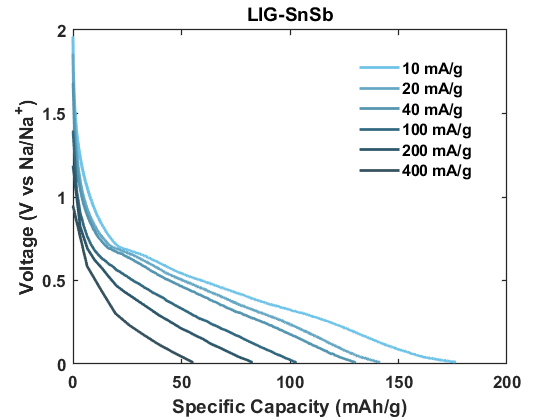


Figure S12 Discharge curve of a) LIG-Sn b) LIG-Sb c) LIG-SnSb at higher current rate in lithium-ion battery half-cell.

Figure S13 Discharge curve of a) LIG-Sn b) LIG-Sb c) LIG-SnSb at higher current rate in sodium-ion battery half-cell.

The discharge curve of LIG-metal composites in Na half-cell are reported in Figure S13. The LIG-Sn still displays the best performances, however, at the higher current rate the capacity goes to zero. While LIG-SnSb still displays at 400 mA g^-1^ a residual capacity of almost 50 mAh g^-1^. LIG-Sb electrode on the other hand, was cycled only at 10, 20 and 40 mA g^-1^ in order to prevent the degradation of the electrode observed in other cells at higher current rates.

1. Ferrari AC. Raman spectroscopy of graphene and graphite: Disorder, electron–phonon coupling, doping and nonadiabatic effects. *Solid State Commun*. 2007;143(1-2):47-57.

2. Liu F, Lee CW, Im JS. Graphene-Based Carbon Materials for Electrochemical Energy Storage. *J Nanomater*. 2013;2013. doi:10.1155/2013/642915

3. Scaravonati S, Sidoli M, Magnani G, et al. Combined capacitive and electrochemical charge storage mechanism in high-performance graphene-based lithium-ion batteries. *Mater Today Energy*. 2022;24:100928.

1. *Corresponding author. Tel. +39 0521 905282. E-mail: [daniele.pontiroli@unipr.it](mailto:daniele.pontiroli@unipr.it) (Daniele Pontiroli) [↑](#footnote-ref-1)
